# Mapping and determinism of soil microbial community distribution across an agricultural landscape

**DOI:** 10.1002/mbo3.255

**Published:** 2015-04-01

**Authors:** Florentin Constancias, Sébastien Terrat, Nicolas P A Saby, Walid Horrigue, Jean Villerd, Jean-Philippe Guillemin, Luc Biju-Duval, Virginie Nowak, Samuel Dequiedt, Lionel Ranjard, Nicolas Chemidlin Prévost-Bouré

**Affiliations:** 1 INRA, UMR1347 AgroécologieBP 86510, F-21000, Dijon, France; 2 INRA, UMR1347 Agroécologie-Plateforme GenoSolBP 86510, F-21000, Dijon, France; 3 Université de Bourgogne, UMR1347 AgroecologieBP 86510, F-21000 Dijon, France; 4 INRA, US1106 InfoSolF-45075, Orleans, France; 5 INRA, UMR1121 Universite de Lorraine (Ensaia)F-54518, Vandoeuvre-les-Nancy, France; 6 AgroSup Dijon, UMR1347 AgroecologieBP 86510, F-21000, Dijon, France

**Keywords:** Agricultural practices, bacterial diversity, environmental filters, landscape, mapping, soil microbial ecology

## Abstract

Despite the relevance of landscape, regarding the spatial patterning of microbial communities and the relative influence of environmental parameters versus human activities, few investigations have been conducted at this scale. Here, we used a systematic grid to characterize the distribution of soil microbial communities at 278 sites across a monitored agricultural landscape of 13 km². Molecular microbial biomass was estimated by soil DNA recovery and bacterial diversity by 16S rRNA gene pyrosequencing. Geostatistics provided the first maps of microbial community at this scale and revealed a heterogeneous but spatially structured distribution of microbial biomass and diversity with patches of several hundreds of meters. Variance partitioning revealed that both microbial abundance and bacterial diversity distribution were highly dependent of soil properties and land use (total variance explained ranged between 55% and 78%). Microbial biomass and bacterial richness distributions were mainly explained by soil pH and texture whereas bacterial evenness distribution was mainly related to land management. Bacterial diversity (richness, evenness, and Shannon index) was positively influenced by cropping intensity and especially by soil tillage, resulting in spots of low microbial diversity in soils under forest management. Spatial descriptors also explained a small but significant portion of the microbial distribution suggesting that landscape configuration also shapes microbial biomass and bacterial diversity.

## Introduction

Soil microorganisms are the most abundant and diverse living organisms on earth and are key players in the biogeochemical cycles. However, the environmental factors shaping soil microbial community abundance and assembly are still unclear, which limits our understanding of the role of soil biodiversity in ecosystem services (Gardi et al. 2009). Since the 18^th^ century, ecologists studying macroorganisms have often used spatial approaches to better understand the processes and filters which drive the magnitude and the variability of biodiversity (Martiny et al. 2006). More recently, microbial ecologists have found that these approaches can also be applied to soil microorganisms (Ettema and Wardle 2002). Consequently, the number of soil microbiology studies integrating a spatial dimension has increased considerably during the last decade. These studies have systematically demonstrated a significant spatial structuring of microbial communities over multiple spatial scales (i.e., that the high spatial variability/heterogeneity of microbial community characteristics is not randomly distributed in space), such as: the microscale (from *μ*m^2^ to mm^2^, Nunan et al. 2003), plot scale (from m^2^ to hundreds of m^2^; Rousk et al. 2010), regional scale (from km^2^ to hundreds of km^2^, Dequiedt et al. 2009; Drenovsky et al. 2010) and global scale (ca. >100 000 km^2^; Dequiedt et al. 2011; Griffiths et al. 2011; Fierer and Ladau 2012; Serna-Chavez et al. 2013). All these scales are relevant to better understand the ecology of soil microorganisms and the determinism of their diversity as they represent the multiple levels of spatial heterogeneity in the soil matrix, climatic conditions, geomorphology, and land use that drives soil microbial diversity (Ettema and Wardle 2002).

Even if the sets of environmental variables were not always completely similar among the studies at the different spatial scales, each of them allowed the identification of environmental filters shaping soil microbial communities. On a broad scale, environmental filters involved in the distribution of microbial communities were identified as soil type, with a significant effect of pH, carbon content and soil texture, as well as of additional factors such as land use and climatic conditions (Fierer and Jackson 2006; Bissett et al. 2010; Pasternak et al. 2013). At the soil microscale, factors such as porosity (Chenu et al. 2001) conditioning carbon substrate and nutrients availability as well as the level of protection of aggregates for microorganisms to surrounding perturbations (Constancias et al. 2013), were identified as drivers of microbial community variation between the different microhabitats. At the intermediate plot scale, proximal factors such as pH (Rousk et al. 2010), organic carbon content (Saetre and Bååth 2000), texture, and land management (Philippot et al. 2009) have been highlighted as important drivers. Altogether, these studies suggested that although similar environmental drivers are involved in shaping microbial communities at every scale, particular filters may have a significant influence at a particular scale. In this context, it is now crucial to investigate an up scaling approach and provide a generic response to the question: which filter for which scale?

Considering the added complexity of shaping soil microbial diversity while up scaling, a gap remains in our knowledge of community distribution at the landscape scale, that is, intermediate between the plot and territory scales. This scale is relevant since it may integrate a strong variability in soil types potentially close to that of a region and because it is the scale of human activities at which land use and agricultural practices are integrated. Microbial investigations at this particular spatial scale are rare and have focused on particular homogeneous ecosystems in terms of land management. Zinger et al. (2011) focused on Alpine natural ecosystems to decipher the influence of plant cover, soil physicochemistry and space in determining soil microbial communities. Other studies focused on an agricultural landscape, but were limited to a restricted mosaic of experimental plots and did not integrate landscape variability or spatial configuration (Enwall et al. 2010; Wessén et al. 2011). Altogether, they have highlighted the need for investigations on a landscape scale to better understand the impact of land management versus soil physicochemical characteristics on indigenous microbial communities. Landscape is also the scale for human activities and decision makers, and a deeper understanding of the relative influence of land use and habitat heterogeneity on below ground soil diversity could be helpful to formulate management strategies for a sustainable land use.

The present study was designed to map and characterize the spatial variation of the soil microbial community across a landscape and to rank the environmental and land use filters influencing this distribution. The studied landscape consisted of forest and arable plots under various types of agricultural management. Soils (*n* = 278) were sampled within a systematic sampling grid (spacing of 215 m) covering the entire landscape (13 km^2^). Physicochemical characteristics and the type of land management were precisely referenced for each soil. Soil molecular microbial biomass was determined from the DNA yield of each soil sample (Dequiedt et al. 2011) and bacterial diversity by massive inventory of the 16S rRNA gene sequences amplified from this soil DNA. Geostatistical approach was used to explain the spatial variability in microbial abundance and diversity and to provide prediction maps. The relative contributions of land management, soil physicochemical characteristics, and space in determining microbial abundance and bacterial diversity distribution were identified and ranked by variance partitioning. We hypothesized that land management, especially agricultural practices, would be the main drivers of microbial abundance and diversity at the landscape scale, due to smaller variations of soil physicochemical characteristics than at wider scales. Spatial descriptors were also integrated into the analysis to better decipher their relative contributions to community variation across landscape and to consider other neutral processes in community distribution.

## Experimental Procedures

### Site description, sampling strategy, and data collection

The study was carried out on a monitored landscape covering 13 km^2^ in Burgundy (France, Lat: 47°14′N, Long: 5°03′E), characterized by oak hornbeam deciduous forests (3.86 km^2^) and intensive agricultural croplands (9.22 km^2^). The site is under continental climate, with a mean annual air temperature of 10.4°C and a mean annual rainfall of 762 mm (period 1968 – 2011). The whole area is situated on deep calcisol (IUSS Working Group, WRB 2006) of mainly silty or silty clay texture and is slightly sloping. Croplands were planted with winter crops (winter wheat, oilseed rape) in rotation with late sown crops (spring barley). Crop species and management practices were recorded from 2004 to 2011 over the whole study area.

The sampling design covers the entire landscape and is based upon a square grid with spacing of 215 m which corresponds to 248 sites. It also includes 30 additional observations positioned randomly within the grid, which permit exploration of the variation over distances less than 215 m (10–100 m from the closest site). All sites were sampled in September 2011. At each site, five soil cores (core diameter: 5 cm; 0–20 cm depth) were collected on a surface of 4 m^2^ at inter row for agricultural sites and at least 1 m away from trees, then bulked and sieved through 2-mm mesh. Samples were lyophilized at −80°C and stored at −40°C in the soil conservatory of the GenoSol platform (http://www2.dijon.inra.fr/plateforme_genosol). Samples were randomized prior to analysis to avoid batch effects. Physicochemical analyses (pH, organic carbon, total nitrogen, CaCO_3_ and texture) were performed as described by Dequiedt et al. (2011). Soil organic carbon was determined by loss on ignition method (https://www6.lille.inra.fr/las/Methodes-d-analyse/Sols/04.-Carbone-Azote-Matieres-Organiques/SOL-0402-Perte-au-feu-a-1100-C.).

### Molecular characterization of soil microbial communities

#### Soil DNA extraction, quantification and purification

DNA was extracted and purified from the 278 soil samples using the GnS-GII procedure as described by Plassart et al. (2012). Crude DNA extracts were quantified by agarose gel electrophoresis stained with ethidium bromide and using calf thymus DNA as standard curve, reported to be reliable for estimating microbial biomass in Dequiedt et al. (2011). Crude DNA was then purified using a MinElute gel extraction kit (Qiagen, Courtabeoeuf, France) and quantified using QuantiFluor staining kit (Promega, Madison, Wisconsin, USA), prior further investigations.

#### PCR amplification and pyrosequencing of 16S rRNA gene sequences

Amplification targeted the 16S rRNA V3-V4 gene region using primers F479 and R888 and a nested PCR strategy to add an 10-bp multiplex identifier (MID) barcode, as initially described by Plassart et al. (2012). Equal amounts of each sample were pooled, and all further steps (adapter ligation, emPCR and 454-pyrosequencing) were carried out by Beckman Coulter Genomics (Danvers, MA, http://www.beckmangenomics.com/) on a 454 GS-FLX-Titanium sequencer (Roche, Basel, Switzerland). The raw data sets are publicly available in the EBI database system (in the Short Read Archive) under project accession no. PRJEB5219.

#### Bioinformatics sequence analysis

The bioinformatics analyses were performed using the GnS-PIPE at the GenoSol platform Terrat et al. (2012). Sequences obtained after an initial quality filtering step (>350 bp, no base ambiguity), were aligned with Infernal alignments using a secondary structure of the 16S rRNA gene (Cole et al. 2009), and clustered at 95% sequence similarity into operational taxonomic units (OTU). Clustering was done with a custom PERL program that does not take into account differences in homopolymer length, which can constitute one of the major 454 sequencing errors (Balzer et al. 2011). Procedure details are provided in Table S1. A subsample of 10,800 quality sequences for each sample was randomly selected to allow rigorous comparison of the data. Bacterial diversity was characterized by OTU richness, evenness, and Shannon index (Haegeman et al. 2013).

### Metadata analysis

#### Clustering of land cover and agricultural practices into land management categories

In order to summarize the land management practices over the entire landscape, a factor analysis for mixed data was used to define land management clusters using the *FactoMineR* package (Lê et al. 2008) with input data such as land use, soil tillage, crop rotation diversity (number of plant types in the crop rotation), pesticide treatment frequency index.

#### Interpolated mapping

A geostatistical method was used to map physicochemical data and microbial communities and to characterize their spatial variations. As the studied variables do not follow a required Gaussian distribution, they were first transformed using the non parametric rank order (or normal scores) transformation prior to considering the spatial correlations (Juang et al. 2001). Conventionally in geostatistical analysis, an estimate of a variogram model is computed based on the observations which describe the spatial variation of the property of interest. This model is then used to predict the property at unsampled locations using kriging (Webster and Oliver 2007). A usual method for variogram estimation is first to calculate the empirical (so called experimental) variogram by the method of moments (Matheron 1965), and then to fit a model to the empirical variogram by (weighted) nonlinear least squares. We investigated also an alternative method which uses maximum likelihood (ML) to estimates parameters of the model directly from the data, on the assumption that it is a multivariate normal distribution. We retained the Matérn model which can describe various spatial processes (Minasny and McBratney 2005). The validity of the fitted geostatistical model was assessed in terms of the standardized squared prediction errors (SSPE) using the results of a leave one out cross-validation. If the fitted model is a valid representation of the spatial variation of the soil or microbial property, then these errors have a *χ*^2^ distribution which has a mean of 1 and median 0.455 (Lark 2002). The mean and median values of the SSPE were also calculated for 1000 simulations of the fitted model to determine the 95% confidence limits. The ordinary kriging estimation was performed in the standardized rank space and then the kriging estimates were back transformed into the original space. We used the geostatistical analysis gstat and GeoR R package for variograms analysis and kriging (Ribiero and Diggle 2001).

#### Variance partitioning

**Figure 1:**
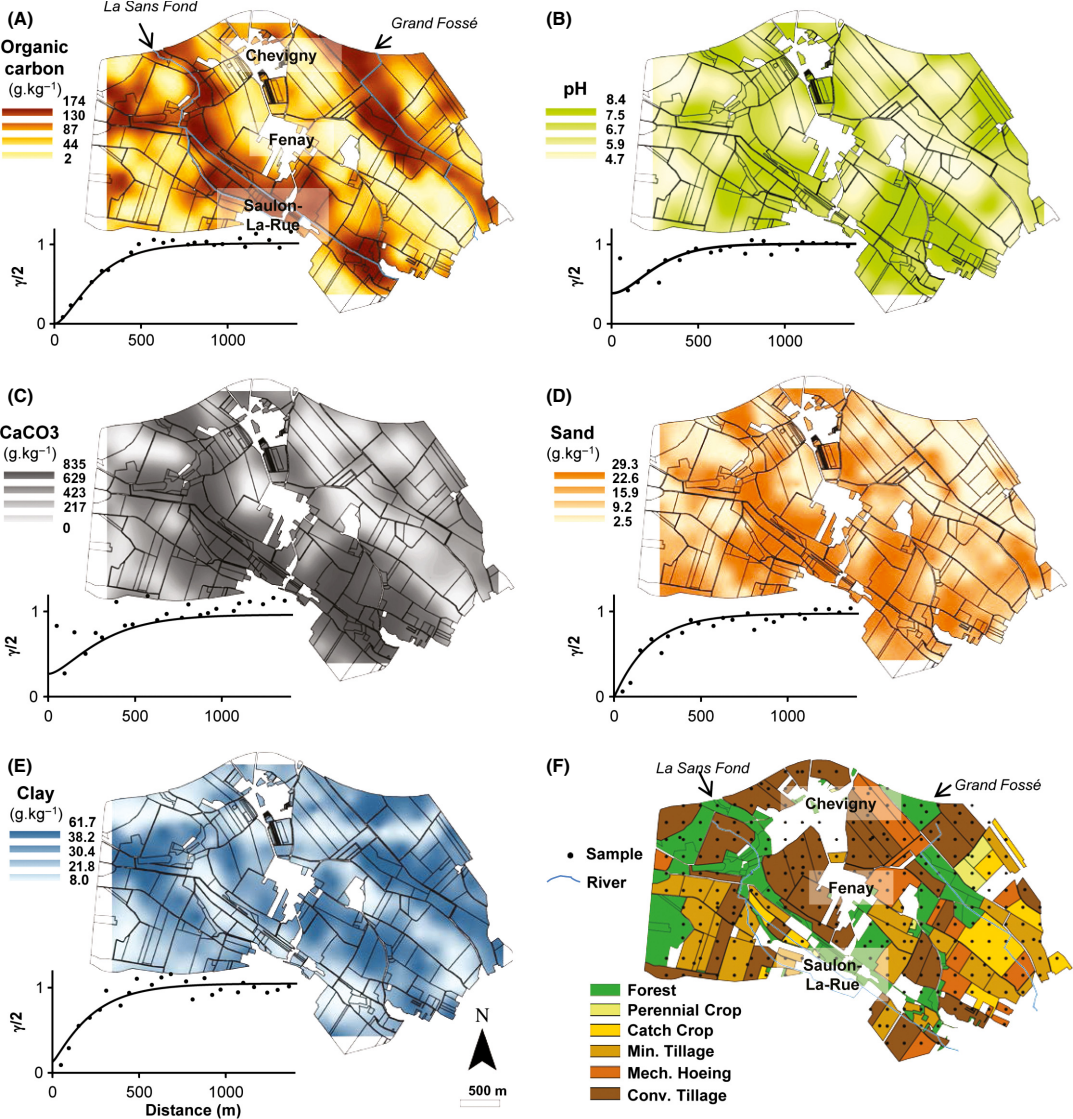
Maps and variogram soil and land use characteristics observed at the scale of the Fénay Landscape. Map of (A) soil organic carbon content, (B) soil pH, (C) CaCO_3_, (D) sand content, (E) clay content, and (F) land management clusters. Points indicate the sampling locations. Min., minimum; mech., mechanical; conv., conventional. For each kriged map the color scale to the left of each map indicates the extrapolated values expressed as g.kg^−1^ of sample excepted for pH. Points represent the experimental variogram, continuous lines the Matérn models fitted by maximum likelihood method. Geostatistics and cross-validation parameters are provided in Table S2.

The relative contributions of soil physicochemical parameters, land management (Fig.1), and space in shaping the patterns of soil microbial abundance and bacterial diversity were estimated by variance partitioning. A Principal Coordinates of a Neighbour Matrix approach (PCNM) was used to describe and identify the scales of spatial relationship between samples (Dray et al. 2006). This PCNM method was applied to the geographic coordinates and yielded 76 PCNM with significant Moran index (*P *<* *0.001), representing the spatial scales that the sampling scheme could perceive (Ramette and Tiedje 2007). The spatial neighborhood described by each PCNM was determined from Gaussian variogram models (Bellier et al. 2007). All quantitative (response and explanatory) data were standardized in order to have an approximated Gaussian and homoskedastic residual distribution. To determine the environmental parameters significantly shaping bacterial communities, a stepwise selection procedure was first applied to all physicochemical and land management variables by maximizing the adjusted *r*^2^ while minimizing the Akaike Information Criteron (Ramette 2007). Spatial descriptors were then selected from the model residuals (Brocard et al., 2004). These selection steps were done to limit over fitting and to exclude co linear variables (Ramette 2007). The respective amounts of variance (i.e., marginal and shared) were determined by canonical variation partitioning and the adjusted *r*^2^ with RDA (Ramette 2007) for microbial biomass, bacterial richness, evenness, and Shannon's diversity index. The statistical significance of the marginal effects was assessed from 999 permutations of the reduced model. All these analyses were performed with R using the vegan package (Oksanen et al. 2011). All these analyses were performed with the R free software (http://www.r-project.org/).

## Results

### Landscape variability and distribution of environmental characteristics

Most soils (70%) in the studied landscape were silty (median 56.7%) or clayey (median 34.3%) with alkaline pH (median 8.0, Table1). Organic carbon and total nitrogen contents were highly correlated (*r*^2^ = 0.92, *P *<* *0.001) and ranged from 1.74 to 174 and 0.835 to 14.6 g.kg^−1^, respectively (Table1). Geostatistical mapping of the environmental variables revealed a heterogeneous distribution of soil characteristics across the landscape, which was spatially structured (Fig.1). High values of soil organic carbon content were systematically found under forest and in agricultural plots beside the “*La Sans Fond*” and “*Grand Fossé*” rivers (Fig.1A). Acidic soils were mainly located at the north east of “*Fénay*” village and in the western part of the studied area. Alkaline soil zones were found near the “*La Sans Fond*” river (Fig.1B), together with high sand and CaCO_3_ contents (Fig.1C–E). The validity of the spatial predictions of soil characteristics was confirmed by the results of the cross-validation. All the indicators (median and mean of the SSPEs) fall within the 95% confidence intervals (Table S2). The fitted models gave effective ranges from 611 to 839 m, depending on the soil parameters (Fig.1, Table S2), indicating that all soil characteristics were spatially structured in patches of several hundreds of meters.

**Table 1:** Summary statistics of soil characteristics (*n* = 278)

	Mean (SD)	Median	[min; max]
Physicochemical			
Organic carbon (g.kg^−1^)	21.9 (15.8)	17.1	[1.7; 174]
Total nitrogen (g.kg^−1^)	2 (1.3)	1.6	[0.8; 14.6]
C:N ratio	10.7 (1.6)	10.4	[1; 22.2]
pH	7.7 (0.7)	8.0	[4.7; 8.4]
CaCO_3_ (g.kg^−1^)	84.6 (161.2)	3.3	[0; 835]
Clay (%)	33.3 (9.5)	34.3	[8; 61.7]
Silt (%)	57.9 (9.6)	56.7	[35.5; 86.2]
Sand (%)	8.8 (4.8)	7.4	[2; 29.3]
Microbial characteristics			
Microbial biomass	65.2 (55.9)	48.5	[2.28; 372.0]
Bacterial richness	1276.2 (145.3)	1262.0	[850; 1761.0]
Bacterial evenness	0.8 (0.02)	0.8	[0.7; 0.8]
Bacterial Shannon index	5.5 (0.2)	5.5	[4.5; 6.1]

Land use and agricultural practices were clustered into six categories (from forest to agricultural plots with a gradient of cropping intensity; see materials and methods) and mapped across the landscape. Six clusters were identified and discriminated first by land cover (forest vs. agricultural plots), secondly by soil tillage intensity (no tillage, minimum tillage, mechanical hoeing, conventional tillage) and finally by the presence of a catch crop. The pesticide treatment frequency index and crop rotation diversity (number of plant types) were not discriminating. These clusters followed a gradient in cropping intensity and in the diversity and persistence of plant cover, that is, Forest (forest, no tillage, no catch crop, *n* = 44); Perennial crop (three frequently mowed grasslands, three blackcurrant and 1 Miscanthus, *n* = 7); Catch crop (agricultural plot, minimum tillage, catch crop, *n* = 22); Minimum tillage (agricultural plot, minimum tillage, no catch crop, *n* = 57); Conventional tillage (agricultural plot, conventional tillage, no catch crop, *n* = 104); Mechanical hoeing (agricultural plot, mechanical hoeing, no catch crop, *n* = 33).

Agricultural plots in the conventional tillage and mechanical hoeing clusters were mainly situated between the villages of “Chevigny” and “Fénay” whereas most plots in the minimum tillage cluster (with or without catch crop) were found to the extreme south west and south east. The forests plots were mainly situated beside the two rivers (“La Sans Fond” and “Grand Fossé”, Fig.1F).

### Landscape distribution of molecular microbial biomass

**Figure 2:**
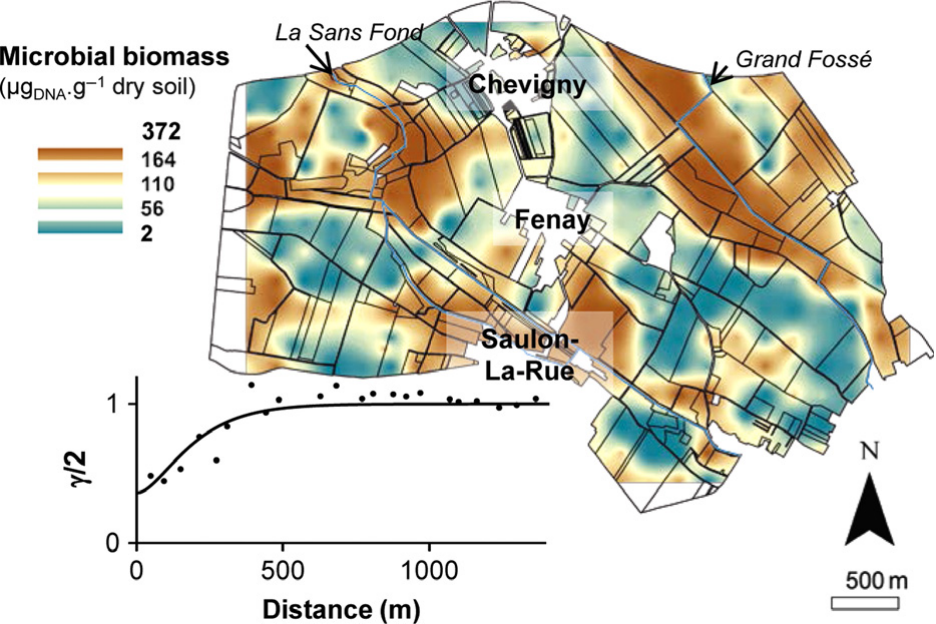
Map and variogram of soil molecular microbial biomass observed at the scale of the Fénay landscape. The color indicates the extrapolated values expressed as *μ*g of DNA.g^−1^ of soil sample. Points represent the experimental variogram, and continuous lines the Matérn models fitted by maximum likelihood method. Geostatistics and cross-validation parameters are provided in Table S2.

The amount of DNA recovered from the 278 soils of the landscape ranged from 2.28 to 372.0 *μ*g DNA.g^−1^ dry soil (Table1). The mean recovery was 65.2 *μ*g DNA.g^−1^dry soil with most soils (90%) yielding concentrations below 126 *μ*g DNA.g^−1^dry soil. The map of microbial biomass highlighted its heterogeneous distribution and revealed high values under forest and under agricultural plots close to the “*Grand Fossé*” river, at the west of “*Chevigny*” and “*Saulon-La-Rue*” and at the extreme east of the Fénay landscape (Fig.2A). The validity of the spatial prediction is confirmed by the cross-validation results (Table S2). The fitted model gave an effective range of 521 m (Fig.2, Table S2) confirming the spatial structure of microbial biomass in patches of several hundreds of meters across the Fénay landscape. Moreover, the small value of the *ν* parameter indicated a rough spatial process over small distances (Table S2).

### Landscape distribution of bacterial diversity

Pyrosequencing of 16S rRNA genes yielded a total of 5.10^6^ sequences (10,800 quality sequences per sample). The rarefaction curves of bacterial OTU confirmed that our sequencing effort allowed a fine description of the bacterial diversity in each soil sample (data not shown). Bacterial richness across the Fénay landscape ranged from 850 to 1,761 OTU with a mean of 1,276 OTU (Table1). Most soils (85%) exhibited a bacterial richness between 1,100 and 1,480 OTU (Table1). Soil bacterial evenness ranged from 0.64 to 0.83 with a mean of 0.77 and most samples (90%) exhibited an evenness value >0.74. Shannon index ranged from 4.43 to 6.17 with a mean of 5.2. Eighty percent of the soils gave values between 5.3 and 5.9 (Table1).

**Figure 3:**
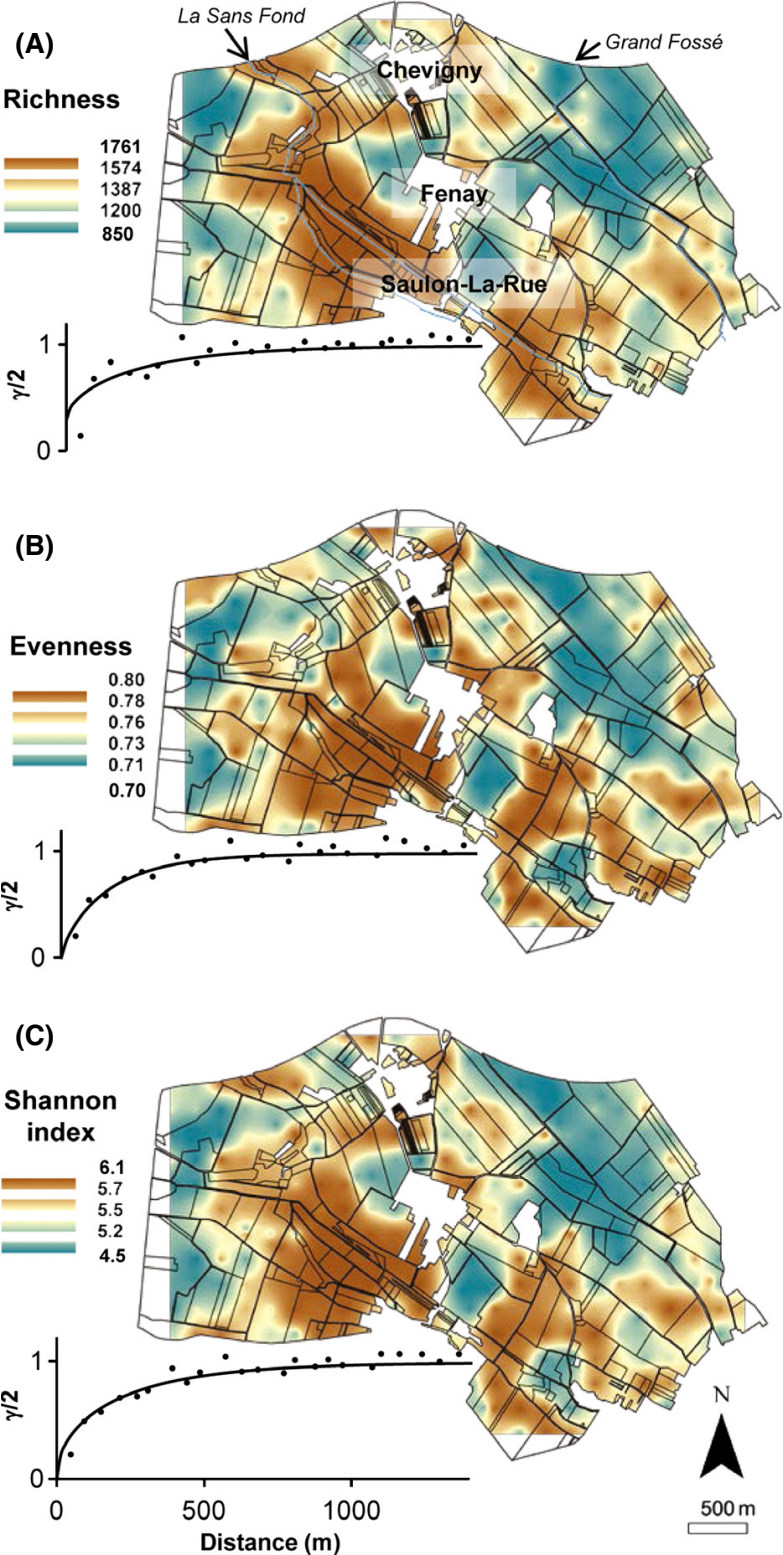
Maps of (A) bacterial richness, (B) bacterial evenness, and (C) bacterial Shannon index parameters measured on the scale of the Fenay landscape. The color indicates the extrapolated values. Points represent the experimental variogram, and continuous lines the Matérn models fitted by maximum likelihood method. Geostatistics and cross-validation parameters are provided in Table S2.

Visual examination of maps of bacterial richness, evenness, and Shannon index evidenced a heterogeneous distribution and broad similar patterns (Fig.3A–C). However, a more precise inspection revealed several differences between bacterial diversity parameters with hotspots of bacterial richness located all along the “*Sans Fond*” river as well as at the east of “*Chevigny*” and “*Saulon-La-Rue*” villages (Fig.3A). Bacterial evenness was distributed in more numerous and smaller patches than bacterial richness, with high values located between the “*Fénay*” and “*Saulon-La-Rue*” villages and cold spots in the north east of “*Chevigny*” (Fig.3B). The interpolated map of Shannon diversity index showed an intermediate distribution between bacterial richness and evenness with hotspots of diversity along the “*Sans Fond*” river as well as at the east of “*Saulon-La-Rue*”, whereas cold spots were found in the north east of the landscape (Fig.3C).

The results of the cross-validation confirmed the validity of the spatial predictions of the bacterial diversity (Table S2). The fitted Matérn models showed effective ranges of 807 m, 521 m, and 758 m for bacterial richness, bacterial evenness, and Shannon index, respectively (Fig.3, Table S2). The small values of *ν* parameter indicated rough spatial processes of bacterial diversity over a small distance (Table S2).

### Variance partitioning of microbial community

**Figure 4:**
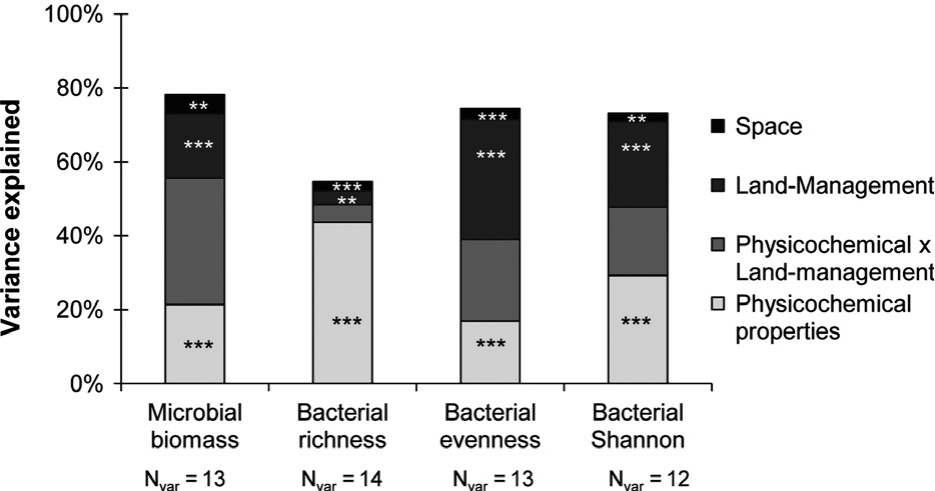
Variance partitioning of molecular microbial biomass and bacterial diversity parameters. The amount of explained variance corresponds to the adjusted *r*^2^ values of the contextual groups using partial redundancy analysis: 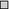 soil physicochemical characteristics; 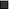 land management 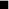 space; 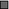 shared amount of variance between soil characteristics and land management that could not be tested. The significance level of the contribution of the sets of variables is indicated as follows **P < 0.01 and ***P < 0.001. N_V__ar_ is the number of explanatory variables retained after selecting the most parsimonious explanatory variables (by minimizing the AIC, akaike information criterion).

The partial regression models demonstrated a systematically significant influence of soil characteristics, land management, and spatial descriptors on microbial biomass and bacterial diversity variation. The total amount of explained variance was 78.1% for microbial biomass, and 54.6%, 74.4%, and 73.1% for bacterial richness, evenness, and Shannon index, respectively (Fig.4). Soil characteristics were the best predictors of microbial biomass (21.4%), bacterial richness (43.7%), and Shannon diversity index (29.3%) whereas land management was the best descriptor of bacterial evenness (32.4%, Fig.4) which was not explained by the spatial variations of the environmental variables. Physicochemical parameters and land management clusters jointly explained a large amount of the total variance (from 4.8% to 34.2%, Fig.4) that could not be tested.

The marginal effects of each filter within the sets of soil characteristics and spatial descriptors were ranked according to their respective amounts of variance explained, and to their standardized estimated coefficients (Table2). For each filter, the marginal effect accounted for relatively small, but significant, proportions of the total variance (from 0.1% to 10%) due to the large number of parameters involved. Regarding the soil characteristics, organic carbon content (10.1%), C:N ratio (1.5%) and clay content (0.5%) were the main drivers of microbial biomass, with organic carbon and clay content having a positive effect (indicated by a positive sign for the standardized coefficient) and C:N ratio a negative effect (Table2). The positive influence of soil organic carbon might be partly explained by the fact that microbial biomass represents a proportion (between 2% and 5%) of soil organic matter. On the other hand, pH, clay, and CaCO_3_ contents were the main drivers of bacterial richness, evenness, and Shannon index (explained variance ranging from 0.8% to 6.1%) with pH and CaCO_3_ having a positive influence and clay content a negative influence (Table2).

**Table 2:** Model parameters for the distribution of microbial biomass and bacterial diversity within the Fénay Landscape

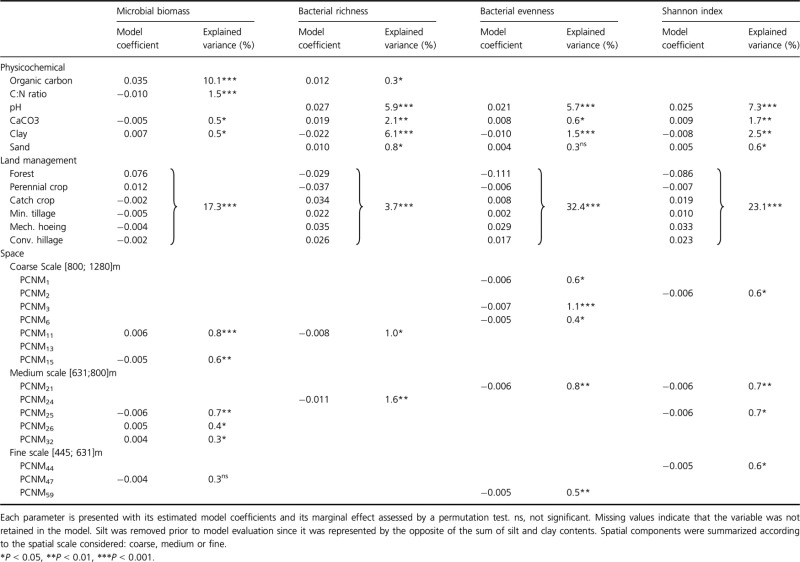

Land management was not included in the filter ranking since it was impossible to determine the relative contributions of each category. However, comparison of the signs and values of the standardized estimated coefficients highlighted a contrasting influence of forest and perennial crops vs. annual crops (Table2). More precisely, increase of the microbial biomass by land management categories followed the sequence: Forest>Perennial crops>Catch Crop≈Conventional tillage>Mechanical Hoeing>Minimum Tillage. An opposite trend was highlighted for bacterial diversity parameters with annual croplands having a positive influence and forest and perennial crops having a negative one (Table2), following the sequence: Mechanical Hoeing>Catch Crop>Conventional Tillage>Minimum Tillage>Perennial Crops>Forest (Table2).

The spatial descriptors of the studied area corresponded to 76 significant PCNM vectors, each representing different spatial scales (coarse, medium, and fine, Table2). The variance explained by spatial descriptors, independently of environmental variables, ranged from 0.3% to 1.6% of the total variance. Spatial descriptors representing coarse and medium scales were mainly involved in microbial biomass and bacterial diversity distribution. Fine scale descriptors were only involved in bacterial evenness and Shannon index. The influence of the scale was ranked, as described above, by comparing the signs of the standardized coefficients. Both positive and negative influences of spatial descriptors were highlighted to explain variations in microbial biomass whereas only negative influences were highlighted for bacterial diversity. Therefore, microbial biomass distribution was mainly explained by coarse (PCNM_11,_ 804 m radius) and medium scales (PCNM_25_ and PCNM_15_, 670 and 630 m radius), the coarse scale having a positive influence and the medium scale a negative one. A negative effect on bacterial richness was also highlighted at the scales of PCNM_11_ (804 m radius) and PCNM_24_ (624 m radius). A larger number of PCNMs were involved in explaining bacterial evenness and Shannon variations, describing coarse scale (PCNM_3_ and PCNM_2_, for bacterial evenness and Shannon, respectively), medium scale (PCNM_21_ for both evenness and Shannon), and fine scale (PCNM_59_ and PCNM_44,_ respectively).

## Discussion

Most recent studies of soil microbial biogeography have highlighted the major contribution of proximal soil characteristics as drivers of microbial community (Fierer and Jackson 2006; Griffiths et al. 2011). However, the considerable soil heterogeneity occurring on a wide scale may mask other drivers associated with human activities, such as agricultural or industrial practices (Fierer and Ladau 2012). Here, we studied microbial distribution across a landscape, which represents the scale of human activities, to better identify and rank environmental versus land management drivers.

The landscape studied was mainly characterized by alkaline silty soils and a mosaic of different types of land management constituted by forest (18% of the area) and agricultural plots with contrasting agricultural practices (82% of the area). The soil characteristics were spatially structured in patches ranging from 600 m to 800 m, which matched the variations in pedological patterns (data not shown) and the distribution of land management categories (Fig.1). Indeed, lower pH and higher organic carbon, nitrogen contents and C:N ratio were recorded under forest as classically observed (Arrouays et al. 2001). Soil characteristics also matched with landscape geomorphology and especially with the “Sans fond” river. Regarding land management, the forest plots were located along the two rivers whereas the agricultural plots distribution did not match with either landscape geomorphology or pedological patterns.

The amount of soil DNA recovered from the 278 soils under study was within the range classically obtained in soil environments with various soil protocols (Plassart et al. 2012). The great range of variations recorded across the landscape was similar to that observed on the French territory scale (Dequiedt et al. 2011), thus supporting the considerable variability of microbial biomass at both local and global scales. Geostatistical predictions of DNA recovery provided the first map of microbial biomass at this scale. As indicated by the variogram model parameters, the heterogeneous distribution of microbial biomass showed significant spatial organization into patches of several hundreds of meters (about 521 m in radius, Fig.2). A similar heterogeneous and spatially structured distribution was observed at both smaller and larger scales with patches ranging from several millimeters at the soil microscale (Nunan et al. 2003), several tens of meters at the plot scale (Berner et al. 2011; several hundreds of kilometers at the territory scale (Dequiedt et al. 2011), to several thousands of kilometers at the earth scale (Serna-Chavez et al. 2013).

Visual comparison of maps of microbial biomass and environmental characteristics suggested that microbial abundance was influenced by both land management and soil characteristics. Microbial biomass hot spots matched with forest plots, and cold spots with croplands, which also corresponded to the distributions of soil organic carbon contents and C:N ratio. Variance partitioning of microbial biomass revealed that soil characteristics were the main drivers, as previously reported on a larger scale (Dequiedt et al. 2011; Serna-Chavez et al. 2013). More precisely, organic carbon content and C:N ratio were the primary drivers influencing microbial biomass with a positive and negative effects, respectively. This is consistent with several reports that organic carbon availability and soil organic matter recalcitrance to degradation by microbes are related to the abundance of microorganisms (Leckie et al. 2004; de Boer et al. 2005). However, a weak influence of clay content was also recorded, which is not consistent with environmental filters hierarchy observed on a broader scale (Dequiedt et al. 2011). This difference might partly be explained by the smaller variation in soil texture measured on our landscape scale, as compared to the French territory scale (Coefficient Variation [CV] = 16.5% vs. 43.6%; respectively) and contrary to the variations in quantity and quality of organic carbon (CV = 72.1% vs. 80.0%, and 37.1% vs. 15.0%, respectively, Ranjard et al. 2013).

Analysis of the marginal effect of land management categories revealed a negative impact of croplands on microbial biomass but not of forests (Table2). This could be due to the high organic matter content of soil under forest management as compared to the low organic carbon content observed in soils under conventional crops (Arrouays et al. 2001). Comparison of the types of agricultural managements revealed differences only between perennial and non perennial crops, thus, confirming the stimulation of microbial abundance under permanent and diversified plant cover (Lienhard et al. 2014). However, no difference in the effects of tillage regime were observed, which contrasts with recurrent reports of a significant loss of microbial biomass with increased soil disturbance (Govaerts et al. 2007; Lienhard et al. 2014). This discrepancy could result from the covariation of tillage regimes with certain soil characteristics in our landscape (e.g., soil organic carbon and texture), which might have increased the amount of variance explained by interaction between land management and soil characteristics, and hampered our evaluation of the impact of particular agricultural practices.

Characterization of bacterial diversity by pyrosequencing of 16S rDNA from soil DNA revealed significant spatial variations in bacterial richness, evenness, and Shannon index across the landscape which were in agreement with other studies covering variations in physicochemical and land management characteristics at similar or broader spatial scales (Nacke et al. 2011; Shange et al. 2012). Geostatistical interpolation showed spatial patterns characterized by patches of 807 m (richness), 521 m (evenness), and 758 m (Shannon index). The maps of bacterial richness and microbial biomass did not match, confirming that microbial abundance and diversity can be influenced by different drivers (Fierer and Jackson 2006; Dequiedt et al. 2009, 2011). These different patterns might be partially related to the contribution of fungi, protozoa, and other eukaryotes to the DNA pool, which may be under the dependence of drivers different from those of bacterial biomass. Hot spots of richness seemed to occur in the vicinity of the “*Sans fond*” river, suggesting a strong influence of landscape geomorphology but also of soil characteristics since the soils all along this river were alkaline with high soil organic carbon and sand contents. Spatial distributions of bacterial evenness and Shannon index were fairly similar to richness but smaller patches were also apparent, suggesting an impact of other environmental filters. Variance partitioning confirmed the different determinisms of richness and evenness, with richness being mainly influenced by soil characteristics and evenness by land management. This is congruent with recent studies evidencing the major effect of soil characteristics on bacterial richness (Lauber et al. 2009; Kuramae et al. 2012; Rodrigues et al. 2013). Our results support that soil characteristics influence the number of species by modulating soil habitat heterogeneity whereas land management mostly influences bacterial population equilibrium by modulating environmental perturbation.

Focusing more precisely on soil characteristics, our study emphasized the overriding effect of pH as a stimulating factor of bacterial community diversity (richness, evenness, and therefore Shannon index) at various spatial scales (Fierer and Jackson 2006; Green and Bohannan 2006; Rousk et al. 2010). Clay content also appeared to be a significant driver of bacterial richness, evenness, and Shannon index variation but had a deleterious effect. Thus, fine textured soil harbored a large microbial biomass, due to its more extensive microhabitats leading to a high carrying capacity, but only a small number of bacterial species, due partly to the reduced heterogeneity leading to a lesser diversity of microbial habitats at the soil microscale (Carson et al. 2010; Chau et al. 2011). In addition, the reduced evenness might result from the increase of competitive exclusion between populations due to the high homogeneity of soil microhabitats. This observation might be also partly explained by the high level of protection provided by fine texture soil for the bacterial community against environmental perturbations (Chenu et al. 2001; Constancias et al. 2013), leading to a decreased population equilibrium through a diminution of selection process between populations (Giller et al. 1998; Bressan et al. 2008).

Independently of other environmental variables, land management accounted for a small proportion (3.7%) of the explained variance for bacterial richness, in agreement with previous reports that bacterial richness is generally poorly impacted by land use (Enwall et al. 2010; Kuramae et al. 2012). Interestingly, bacterial richness was lower in forest soils (mean of 1191 OTUs) than in crop soils (mean of 1297 OTUs), whereas microbial biomass was strongly stimulated under forest (159 *μ*g DNA.g^−1^ soil for forest soils vs. 47 *μ*g DNA.g^−1^ soil for crop soils). A similar and more significant trend was observed in the positive effect of crop soils on evenness and Shannon index (0.73 vs. 0.78 for forest and crop soils evenness, respectively; 5.16 vs. 5.58 for forest and crop soils Shannon index, respectively). These diversity parameters were positively related, in crop soils, to the gradient of increased soil disturbance by tillage. This stimulatory effect of tillage on soil bacterial diversity may be related to the degree of perturbation induced by this agricultural practice (Acosta-Martínez et al. 2010; Lienhard et al. 2014). According to the “hump back” model between biodiversity and the intensity of environmental perturbation, which suggests that the greatest biodiversity is obtained with moderate environmental perturbation due to a diminution in competitive niche exclusion and selection mechanisms occurring between populations (Giller et al. 1998), our results emphasize that crop soils under conventional tillage and mechanical hoeing would correspond to these conditions (Lienhard et al. 2014).

Spatial descriptors, illustrating neighborhood relationships between samples, systematically accounted for the smallest significant contribution to microbial biomass and diversity distributions at coarse (800–1280 m), medium (630–800 m), and fine scales (440–630 m). In agreement with Hanson et al. (2012), the influence of spatial descriptors might be partly related to variations in unmeasured soil characteristics at the medium scale, whereas it might result from landscape configuration at the coarse and fine scales. The coarse scale represents the global distribution of forest vs. crop patches, and the fine scale represents the distribution of individual agricultural plots subjected to particular practices. These results suggest that landscape configuration would be an additional driver of soil microbial biomass and bacterial diversity distribution. This hypothesis is in agreement with Ranjard et al. (2013), who demonstrated the influence of territory heterogeneity and configuration in shaping bacterial diversity turnover. In addition, our analysis revealed a systematically negative effect of spatial descriptors on bacterial diversity, which suggests that landscape configuration might partially affect bacterial diversity by limiting bacterial dispersal. This result supports the hypothesis that the selection and dispersal limitation of microbial populations are not exclusive as suggested by Hanson and Fuhrman (2012).

Altogether, our study provides the first map of microbial biomass and bacterial diversity across an agricultural landscape, and demonstrated the heterogeneous but spatially structured distribution of the microbial community at this scale, mainly driven by proximal filters such as soil characteristics and agricultural practices. Our results therefore confirm that the landscape is an appropriate scale for robust evaluation of the influence of agricultural land management on soil microorganisms. This spatial scale is also shown to be relevant for modifying and improving human activities in the context of a sustainable use of soil resources. Further analyses are now required to measure and link soil microbial activities with microbial diversity and to identify and better define the bacterial groups and their ecological attributes at this scale.

## Supporting Information

**Figure S1.**Physicochemical characteristics measured at each sampling point. **Figure S2.** Microbial community characteristics at each sampling point. **Table S1.** Steps, parameters and database used in the bioinformatics analysis of 16s rRNA sequences obtained by pyrosequencing. **Table S2.** Geostatistics and cross-validation parameters for the physicochemical and microbial data standardized by Gaussian quantile transformation.
