# Supplementary material for: Mapping and determinism of soil microbial community distribution across an agricultural landscape
Source: Microbiologyopen. 2015 Apr 1;4(3):505–17. doi: 10.1002/mbo3.255 (PMC4475391; doi:10.1002/mbo3.255)
Supplement: Supplementary file 1 — Figure S1.Physicochemical characteristics measured at each sampling point. Figure S2. Microbial community characteristics at each sampling point. Table S1. Steps, parameters and database used in the bioinformatics analysis of 16s rRNA sequences obtained by pyrosequencing. Table S2. Geostatistics and cross-validation parameters for the physicochemical and microbial data standardized by Gaussian quantile transformation. [file mbo30004-0505-sd1.docx]

### Table S1. Steps, parameters and database used in the bioinformatics analysis of 16s rRNA sequences obtained by pyrosequencing.

| **STEP** | **PARAMETER** | **TARGETED 16S rRNA GENE** |
| --- | --- | --- |
| **PREPROCESSING** | Length Treshold | 350 |
|  | Number of Ambiguities tolerated | 0 |
|  | Search for proximal primer sequence | Complete and perfect |
|  | Search for distal primer sequence | Perfect, but potentially incomplete |
| **CLUSTERING** | Chosen level of similarity (%) | 95 |
|  | Ignoring differences in homopolymer lengths | Yes |
| **FILTERING** | Chosen clustering similarity threshold (%) | 95 |
|  | Used taxonomic database | SILVA (r114) |
|  | Chosen taxonomic level | *Phylum* |
|  | Similarity or confidence threshold (%) | 90 |
| **HOMOGENIZATION** | Nb of quality reads kept for each sample | 10 800 |
| **TAXONOMY** | Used taxonomic database | SILVA (r114) |
|  | Method or tool of comparison | USEARCH (6.0.307) |
|  | Similarity or confidence threshold (%) | 80 |
| **ANALYSIS** | Chosen level of similarity (%) | 95 |
|  | Ignoring differences in homopolymer lengths | Yes |

**Table S2. Geostatistics and cross-validation parameters for the physico-chemical and microbial data standardized by Gaussian quantile transformation.**

|  | Nugget^a^  ^(C^_0_^)^ | Sill^b^  ^(C^_0_^+C)^ | Effective  Range^c^  (m) | Structural variance^d^  (%) | ν parameter^e^ | mean of SSPE^f^ | Median of SSPE^f^ |
| --- | --- | --- | --- | --- | --- | --- | --- |
| **Physicochemical** | | | | | | | |
| Organic carbon | 0.00 | 1.02 | 640 | 100 | 1.2 | **1.00** | **0.40** |
| Total nitrogen | 0.08 | 1.01 | 624 | 92 | 1.6 | **0.98** | **0.34** |
| C:N ratio | 0.30 | 1.06 | 835 | 72 | 1.1 | **1.03** | **0.35** |
| pH | 0.38 | 1.01 | 647 | 62 | 2.1 | **1.00** | **0.41** |
| CaCO3 | 0.27 | 0.97 | 839 | 72 | 1.1 | **0.98** | **0.37** |
| Clay | 0.13 | 1.05 | 663 | 88 | 0.6 | **1.00** | **0.39** |
| Silt | 0.14 | 1.04 | 664 | 87 | 0.6 | **1.01** | **0.41** |
| Sand | 0.00 | 0.98 | 611 | 100 | 0.5 | **1.02** | **0.40** |
| **Microbial parameters** | | | | | | | |
| Microbial biomass | 0.36 | 1.00 | 521 | 100 | 1.4 | **1.00** | **0.41** |
| Bacterial richness | 0.29 | 0.99 | 807 | 71 | 0.3 | **1.00** | **0.48** |
| Bacterial evenness | 0.00 | 0.98 | 521 | 100 | 0.4 | **1.00** | **0.41** |
| Bacterial Shannon | 0.00 | 0.99 | 758 | 100 | 0.2 | **1.00** | **0.48** |

^a)^ Nugget variance, the magnitude of the discontinuity of the variogram.

^b)^ Sill, the value of the variogram for distances beyond the range of the variogram.

^c)^ Effective range

^d)^ Structural variance, calculated as (C_1_/(C_o_+C_1_) which represents the amount of variance spatially structured.

^e)^ Kappa, the Matérn smooth parameter

^f)^ Mean and median of the cross validation statistic (SSPE), bold values are in the 0.95 confidence interval.

**Supplementary Figure 1.** Physicochemical characteristics measured at each sampling point

a

b

**
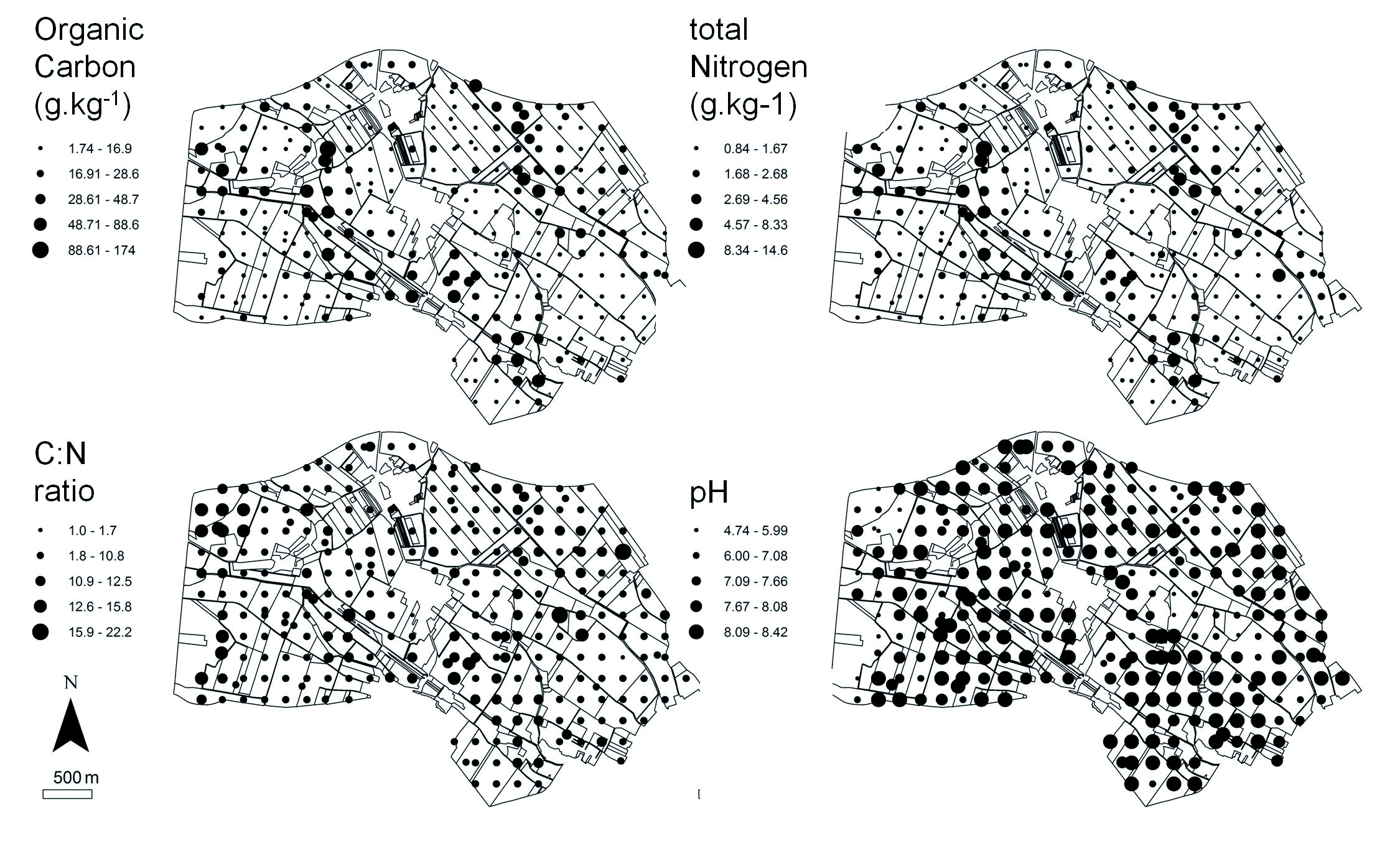

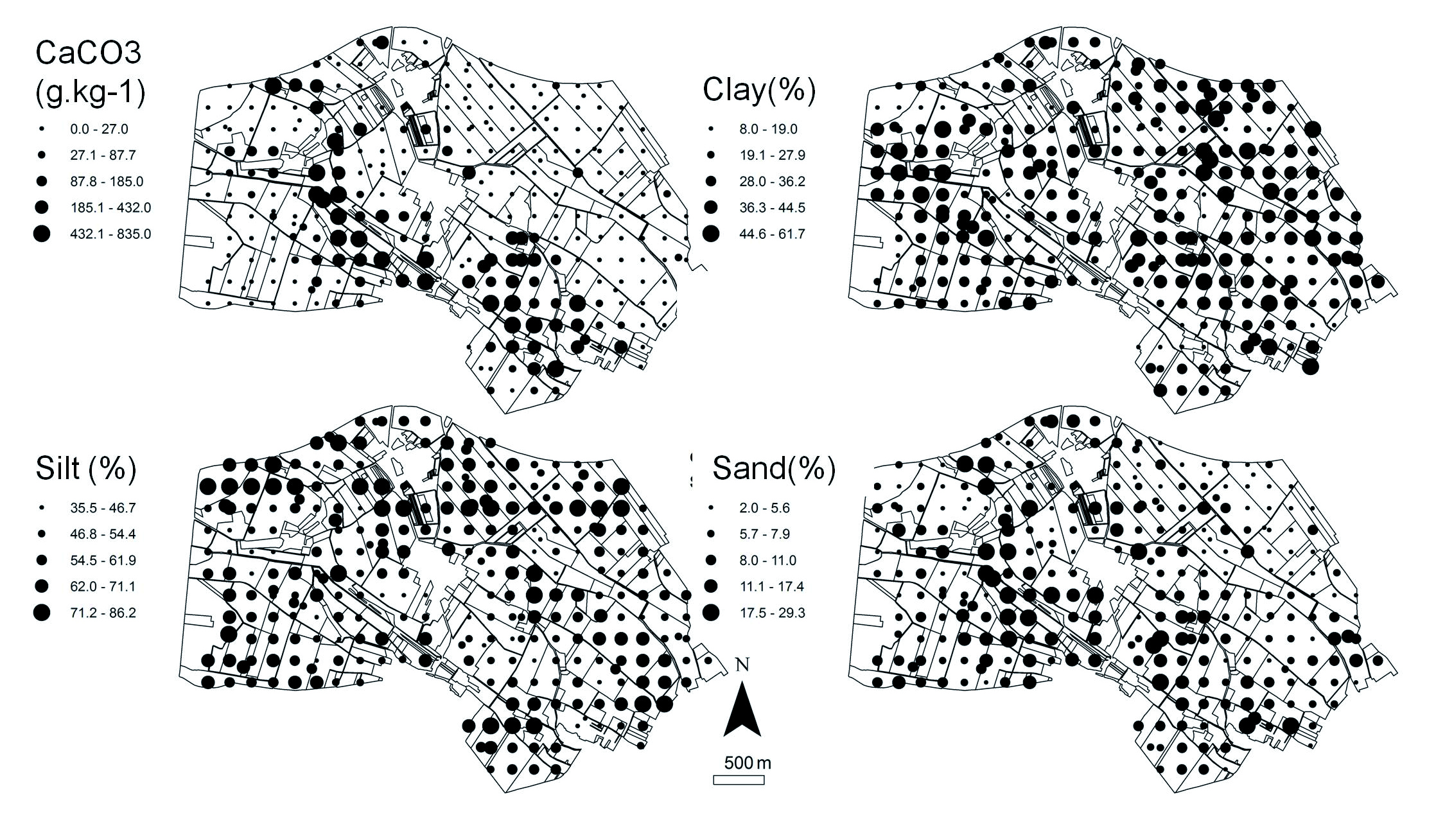
**

c

d

e

f

h

g

Map of (**a**) organic carbon, (**b**) total nitrogen, (**c**) C:N ratio, (**d**) clay, (**e**) silt and (**f**) sand. Circles represent the measured value at each sample point as indicated by the panel nearby each map.

**Supplementary Figure 2.** Microbial community characteristics at each sampling point

**
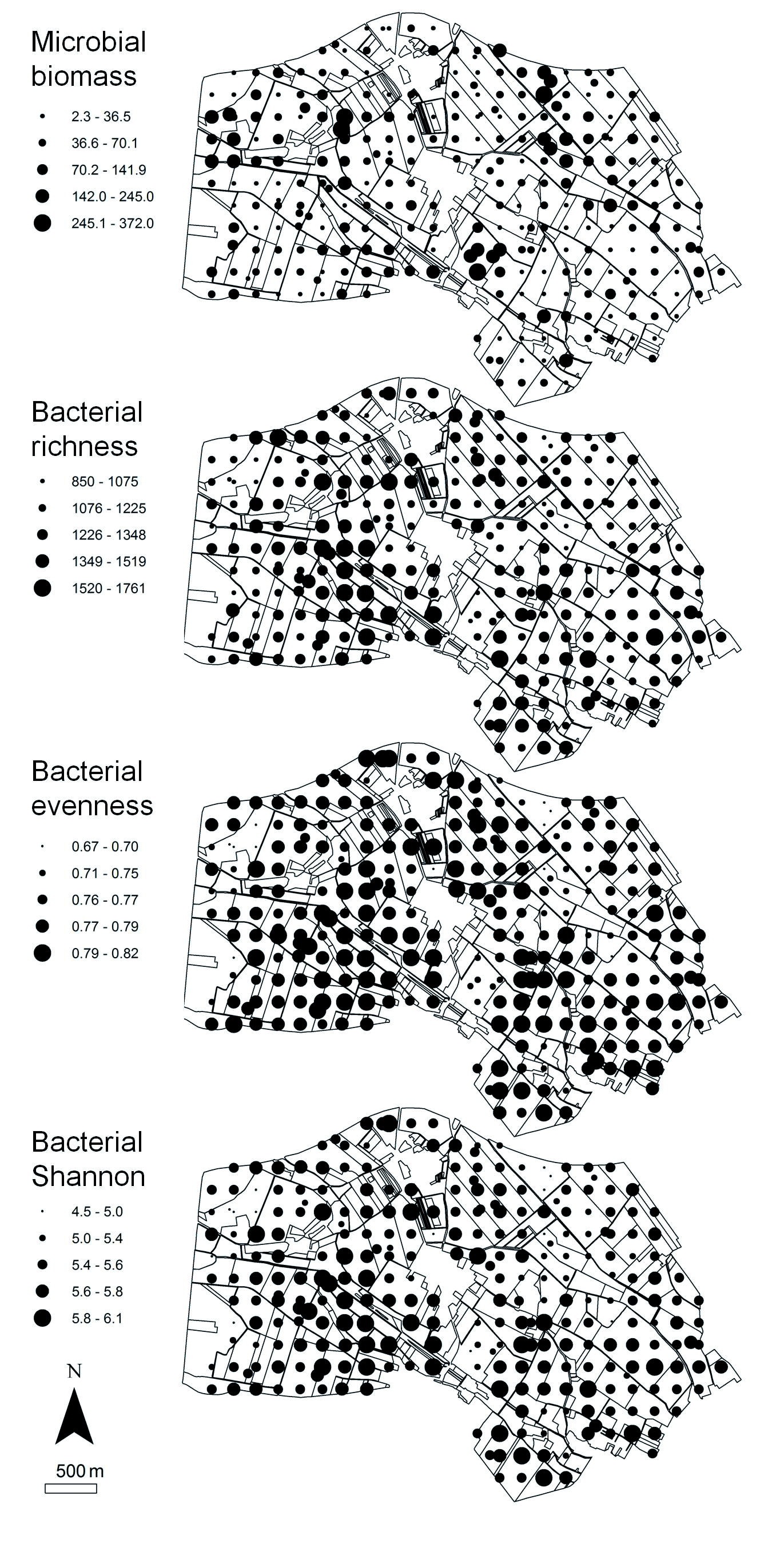
**

a

d

c

b

Map of (**a**) molecular microbial biomass, (**b**) bacterial richness, (**c**) bacterial evenness and (**d**) bacterial Shannon. Circles represent the measured value at each sample point as indicated by the panel nearby each map. Microbial biomass is expressed as µg of DNA.g-1 of dry sample.
